# Determinants of new participation in sports groups among community-dwelling older adults: Analysis of a prospective cohort from The Otassha Study

**DOI:** 10.1371/journal.pone.0275581

**Published:** 2022-10-04

**Authors:** Manami Ejiri, Hisashi Kawai, Yoshinori Fujiwara, Kazushige Ihara, Yutaka Watanabe, Hirohiko Hirano, Hunkyung Kim, Shuichi Obuchi

**Affiliations:** 1 Tokyo Metropolitan Institute of Gerontology, Itabashi-Ku, Tokyo, Japan; 2 Department of Social Medicine, Hirosaki University School of Medicine, Hirosaki City, Aomori, Japan; 3 Gerodontology, Department of Oral Health Science, Faculty of Dental Medicine, Hokkaido University, Sapporo City, Hokkaido, Japan; Universidad Nacional Autonoma de Mexico, MEXICO

## Abstract

Participation in sports groups has health benefits for older adults, such as preventing functional limitations and social isolation. Encouraging participation in sports groups may be an important means of health promotion in older adults. However, there is insufficient research on the determinants of new participation in sports groups to consider effective interventions to promote participation in these groups. We investigated this using data from a 1-year prospective study. Data were obtained from “The Otassha Study” that assessed a cohort of community-dwelling older adults living in an urban area of Japan. Of 769 older adults who participated in a baseline health survey in 2018, 557 participated in a follow-up survey in 2019. We excluded 184 individuals who already participated in sports groups at baseline and 36 with missing data. Participation in sports groups was defined as that occurring more than once a week. Logistic regression analysis was used to identify the determinants of new participation in sports groups, with sociodemographic factors, lifestyle habits, physical functions, cognitive functions, psychological factors, and social factors as independent variables. Forty-one (12.2%) individuals participated in sports groups at follow-up. In the multiple adjusted logistic regression model, new participation in sports groups was significantly associated with female sex (odds ratio [OR] = 5.57, 95% confidence interval [CI]: 1.61‒19.26), engagement in regular exercise (OR = 2.23, 95%CI: 1.03‒4.84), and having a large social network (OR = 1.12, 95%CI: 1.04‒1.20). Physical functions were not associated with new participation. Determinants of new participation were lifestyle habits and social factors, rather than physical functions. Intervention through social networks may be effective in encouraging new participation in sports groups, which, in turn, may facilitate healthy aging.

## Introduction

In aging societies, the extension of healthy life expectancy among older adults is important. Physical activities, including exercise and participation in sports, help prevent functional dependence and reduce mortality [1]. Notably, there is increasing evidence that participation in sports groups results in good health outcomes among older adults. Recent cross-sectional studies have shown that older adults who participated in sports groups had higher self-rated health [2, 3], lower fall rates [4], and more remaining teeth [5] than those who did not participate. Another study reported that regular group exercise contributed to balanced health, including physical, mental, and social well-being [6]. Moreover, longitudinal studies revealed that participation in sports groups was effective in preventing functional disability [7, 8] and social isolation [9] among community-dwelling older adults. In addition to the health benefits described above, engaging in exercise and sports with others, rather than alone, can also help sustain exercise habit. Bandura’s social cognitive theory states that behavior is shaped by the interaction of individual and environmental factors, and emphasizes the importance of social support in relation to behavior [10]. Playing sports with others provides social support, which further increases participation in sports [11]. A previous review also reported that exercise habit is more likely to be sustained in a group than alone [12]. Accordingly, encouraging participation in sports groups can be a crucial means of health promotion among older adults, in addition to formal healthcare services. Therefore, it is necessary to determine factors related to participation in sports groups and discuss effective interventions focusing on these factors.

However, to date, research on participation in sports, either individually or in groups, has been primarily conducted among adults and adolescents, with limited research among older adults [10]. A previous review on the determinants of participation in group exercise only included studies of adolescents and young adults in the United States or Australia [13]. This review pointed out that only a few demographic and environmental factors were examined in this respect.

In Japan, which is the most aged society in the world [14], some recent studies have focused on older adults and have examined the characteristics of participants in sports groups. Yamakita et al. conducted a cross-sectional study from the perspective of five factors; demographic and biological; psychosocial; behavioral; social and cultural; and environmental factors [15]. They found being female, having a high education level, not being employed, having high self-rated health, and not having current smoking habits were positively associated with regular participation in sports groups. Participating in hobby clubs, senior citizen clubs, or volunteer groups, having emotional support, and perceptions of the availability of parks or sidewalks were associated with a high prevalence of participation in sports groups [15]. Nemoto et al. classified sports groups as a type of private group and conducted a longitudinal study to determine the factors that contribute to new participation in these groups [16]. They showed, using a 2-year study, that older adults who had lower educational attainment and worked full-time were less likely to join private groups, whereas those with better self-rated health and mental well-being and closer relationships with their neighbors were more likely to join private groups. While these studies provided valuable insights, no longitudinal studies have assessed determinants exclusively for participation in sports groups; furthermore, prior studies have not evaluated physical and cognitive functions as these were conducted using mail surveys. Therefore, it would be beneficial to evaluate these functions using longitudinal health examination data.

These functions have been considered independent variables of group participation. For example, in a previous longitudinal study that examined the determinants of volunteer group participation, background characteristics (sex, race, education, and marital status) and resources (physical, cognitive, and social function and social capital) were considered as independent variables [17]. While this previous study assessed engagement in volunteer groups, similar determinants may also potentially affect new participation in sports groups. In examining the determinants of participation in sports groups, understanding background characteristics can help identify intervention targets, and understanding resources can help examine intervention methods. If these factors can be identified, participation in sports groups can be encouraged, which in turn may lead to healthy aging. Therefore, we aimed to identify determinants of new participation in sports groups among Japanese community-dwelling older adults using data from a 1-year prospective study.

## Materials and methods

### Participants

**Fig 1:**
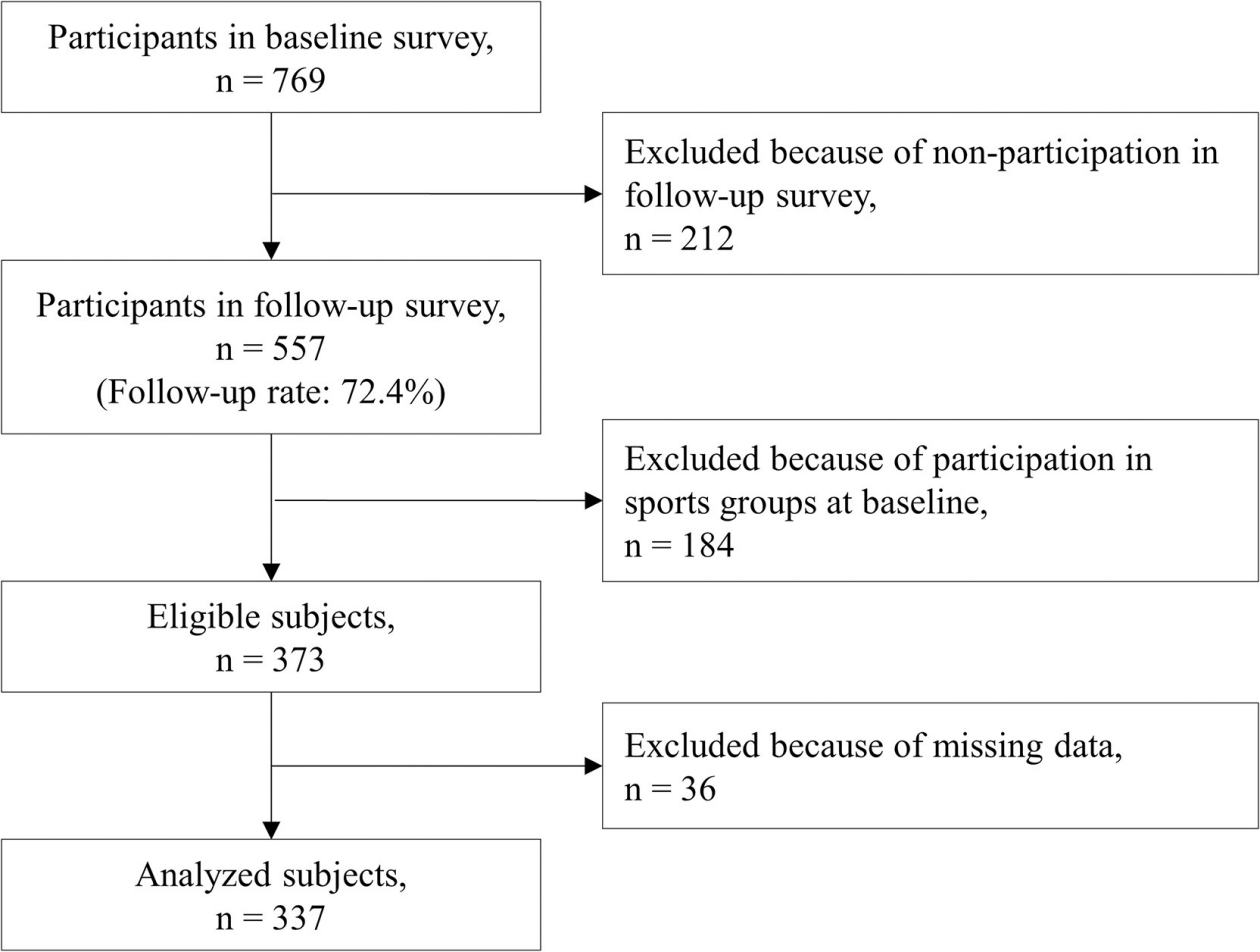
Flow diagram of enrollment of study participants.

Data were obtained from “The Otassha Study,” which included a cohort of community-dwelling older adults living in Itabashi Ward, an urban area of Tokyo, Japan. The Otassha study began in 2011 and involves health checkups every year to date. At the beginning of The Otassha Study, we sent mail recruitment letters to all residents aged 65‒84 years who were registered in the Basic Resident Register as of October 2011, except institutionalized individuals. The respondents participated in comprehensive health checkups that included physical function tests, cognitive function tests, and medical interviews. Follow-up of these participants has occurred every year to date, and new participants have been recruited each year as they turn 65 years old. Details of the checkups are described elsewhere [18]. In the current study, we used data obtained via a baseline (BL) survey in 2018 and a follow-up (FL) survey in 2019. The protocol is the same every year, but the measures vary each year. Because participation in sports groups was queried for the first time in 2018, the baseline for this study was set to 2018. Among 769 individuals who participated in the BL survey, 557 participated in the FL survey (FL rate: 72.4%) (Fig 1). Among those individuals, we excluded 184 who were already participating in sports groups at BL. Thus, 373 participants were included in this study. The definition of participation in sports groups is provided in the Measurement section.

Ethical approval was granted by the ethics committee of the Tokyo Metropolitan Institute of Gerontology (approval no. H16, 2018; H15, 2019). This study was conducted in accordance with the principles of the Declaration of Helsinki. All participants provided written informed consent for participation in both the BL and FL surveys prior to enrollment.

### Measurement

#### Dependent variable

Participation in sports groups was assessed using the following question at both BL and FL: “How often do you participate in a sports group or club?” [15] Based on a previous study that examined the characteristics of older adults who participated in sports groups, those who answered “more than once a week” were considered as “participants in sports groups” and those who answered “one to three times a month,” “several times a year,” or “not participating” were considered as “non-participants in sports groups.” [15] Among those who were non-participants at BL, those who newly participated in sports groups at FL were defined as “new participants in sports groups.”

#### Independent variables

Independent variables included sociodemographic factors, lifestyle habits, physical functions, cognitive functions, psychological factors, and social factors. All variables were collected at BL.

*Sociodemographic factors*. Data on sex and age were obtained from the resident registry system, and those on education status (years of school education) were collected following an interview.

*Lifestyle habits*. Regular exercise was assessed using a questionnaire. It was defined according to the frequency of weekly exercise based on a Japanese nationwide survey, the National Health and Nutrition Survey [19]. Exercising twice a week or more was considered regular, whereas exercising once a week or less was occasional.

*Physical functions*. Hand grip strength (upper body strength) and maximum gait speed (gait speed) were measured. Hand grip strength was measured once using a Smedley-type hand dynamometer (Yagami, Nagoya, Japan). Participants were asked to stand and grip the hand dynamometer as tightly as possible with their dominant hand [20]. The results were presented in kg. Maximum gait speed was measured twice on a 5-m course with a 3-m acceleration and deceleration course placed at each end of the course [20]. Participants were asked to walk as fast as possible, and the faster time of the two trials was used in the analysis. Gait speed at 5 m was calculated as distance (meter) divided by walking time (second).

*Cognitive functions*. The Mini-Mental State Examination was administered by trained psychotherapists [21]. The scores ranged from 0 to 30, with a higher score indicating higher cognitive function.

*Psychological factors*. We assessed self-rated health with the question, “in general, would you say that your health is good?” The answers were categorized as fair or poor.

*Social factors*. The Lubben Social Network Scale-6 (LSNS-6) and “social cohesion and trust” from collective efficacy were measured via a questionnaire. The LSNS-6 measures the number and frequency of contacts with close family and friends [22]. The score ranged from 0 to 30, with a higher score indicating a larger social network. Social cohesion was defined as the willingness to intervene for the common good among neighbors [23]. This scale includes five items scored using a five-point Likert scale. Respondents were asked how strongly they agreed that people around their residential area were willing to help their neighbors [23]. The score ranged from 5 to 25, with higher scores indicating greater social cohesion.

### Statistical analysis

Data on participant characteristics are presented as means and standard deviations for continuous variables and numbers (percentages) of participants for categorical variables. A logistic regression analysis was used to estimate the odds ratios (ORs) and 95% confidence intervals (CIs) of participation in sports groups in two models. Model 1 was a crude model that examined the independent association between each independent variable and participation in sports groups without considering the effect of other variables. Model 2 was a multivariable model that included all independent variables. In each model, “non-participants” was used as the reference category. To investigate the actual determinants of new participation in sports groups among participants with no sports or exercise experience, we conducted a subanalysis excluding participants with regular exercise. Before conducting the research, we calculated the sample size using G*Power 3.1.9.2 [24]. Based on a previous longitudinal study [16], we hypothesized an OR of 1.6 and a new participation rate in sports groups of 13% within 1 year. The total sample size required for logistic regression analysis was 328 (two-tailed, significance level = 0.05, power = 0.8, R^2^ other X = 0.04 [low association]).

All statistical analyses were conducted using IBM SPSS Statistics for Windows, version 23.0 (IBM Japan, Ltd., Tokyo, Japan). The significance level was set at p<0.05.

## Results

The final analysis included 337 participants, and excluded 34 participants with missing data (Fig 1). Among individuals who did not participate in sports groups at BL, 41 (12.2%) became “new participants in sports groups” at FL. The BL characteristics of older adults according to participation status are shown in Tables 1 and 2. Among the new participants in sports groups, 24.4% were men, 34.1% engaged in regular exercise, and 87.8% assessed their own health as fair. Among non-participants in sports groups at FL, 49.0% were men, 21.6% engaged in regular exercise, and 83.1% assessed their own health as fair. The average age was 74.2 years among participants and 72.8 years among non-participants. The average LSNS-6 score was 17.9 among participants and 14.3 among non-participants.

**Table 1:** Baseline characteristics of older adults according to the status of participation in sports groups at follow-up (continuous variables).

	New participants in sports groups (n = 41)		Non-participants in sports groups (n = 296)	
	Mean	(SD)	Mean	(SD)
Age (year)	74.2	(6.85)	72.8	(6.24)
School education (year)	13.8	(2.95)	13.3	(2.92)
Hand grip strength (kg)	26.3	(8.67)	28.9	(8.79)
Maximum gait speed (m/s)	2.1	(0.37)	2.1	(0.46)
MMSE	28.7	(1.21)	28.5	(1.70)
LSNS-6	17.9	(5.41)	14.3	(5.94)
Social cohesion	17.2	(3.40)	17.1	(3.51)

SD, standard deviation; MMSE, Mini-Mental State Examination; LSNS-6, Lubben Social Network Scale-6

**Table 2:** Baseline characteristics of older adults according to the status of participation in sports groups at follow-up (categorical variables).

	New participants in sports groups (n = 41)		Non-participants in sports groups (n = 296)	
	N	%	n	%
Sex				
Male	10	24.4%	145	49.0%
Female	31	75.6%	151	51.0%
Regular exercise				
Regular	14	34.1%	64	21.6%
Occasional	27	65.9%	232	78.4%
Self-rated health				
Fair	36	87.8%	246	83.1%
Poor	5	12.2%	50	16.9%

The ORs and 95% CIs for participation are presented in Table 3. In the crude model of each independent variable (Model 1), women (OR = 2.98, 95% CI: 1.41‒6.29) and those with a higher LSNS-6 score (OR = 1.11, 95% CI: 1.05‒1.18) were significantly more likely to participate in sports groups. In the multiple logistic regression model adjusted for all independent variables (Model 2), women (OR = 5.57, 95% CI: 1.61‒19.26), those engaging in regular exercise (OR = 2.23, 95% CI: 1.03‒4.84), and those with a higher LSNS-6 score (OR = 1.12, 95% CI: 1.04‒1.20) were more likely to participate in sports groups. Physical functions such as hand grip strength and maximum gait speed were not significantly associated with new participation in sports groups.

**Table 3:** Odds ratios (95% confidence intervals) for participation in sports groups at follow-up.

	Model 1	Model 2
	OR (95% CI)	OR (95% CI)
Sex: female (ref.: male)	2.98 (1.41–6.29)	5.57 (1.61–19.26)
Age (years)	1.03 (0.98–1.09)	1.03 (0.97–1.10)
School education (years)	1.06 (0.95–1.18)	1.12 (0.98–1.28)
Regular exercise: Regular (ref.: occasional)	1.88 (0.93–3.79)	2.23 (1.03–4.84)
Hand grip strength (kg)	0.97 (0.93–1.00)	1.03 (0.96–1.11)
Maximum gait speed (m/s)	0.72 (0.35–1.49)	0.82(0.28–2.40)
MMSE (total score)	1.08 (0.87–1.34)	1.04 (0.81–1.34)
Self-rated health: Fair (ref.: poor)	1.46 (0.55–3.91)	1.47 (0.50–4.33)
LSNS-6 (total score)	1.11 (1.05–1.18)	1.12 (1.04–1.20)
Social cohesion (total score)	1.01 (0.92–1.11)	0.95 (0.85–1.05)

Model 1: Crude

Model 2: Adjusted for all independent variables (sex, age, school education, regular exercise, hand grip strength, maximum gait speed, MMSE, self-rated health, LSNS-6, and social cohesion)

OR, odds ratio; CI, confidence interval; ref, reference; MMSE, Mini-Mental State Examination; LSNS-6, Lubben Social Network Scale-6.

The results of the subanalysis that excluded participants with regular exercise also showed that women (OR = 15.1, 95% CI: 2.70‒84.2) and those with a higher LSNS-6 score (OR = 1.16, 95% CI: 1.07‒1.27) were significantly more likely to participate in sports groups (Model 2).

## Discussion

This longitudinal study investigated determinants of participation in sports groups from multiple perspectives, including physical function, cognitive function, and social factors, to clarify effective strategies for promoting participation in these groups. We found that female sex, engaging in regular exercise, and larger social networks were determinants of new participation in sports groups, while physical functions were not.

Evidence regarding demographic factors could help identify subgroups that require a more intensive approach to encourage participation in sports groups. We found that women were 5.5 times more likely to newly participate in sports groups than men. This result was in line with that of a previous cross-sectional study of the characteristics of participants in sports groups [15]. However, it was in contrast to the results of previous studies of physical activity and exercise. According to previous reviews on determinants of physical activity and exercise, men are more likely to engage in physical activity and exercise than women [25–27]. Additionally, a recent Japanese nationwide survey reported that the prevalence of engaging in regular exercise among older adults was higher in men than in women, at 42.9% and 36.5%, respectively [19]. These previous studies reported that older men were more active in sports than women; however, the results of the current study suggest that men do not belong to groups and are rather engaged in solo sports activities. Encouraging men to join sports groups may lead to greater health benefits because exercising with others has been shown to provide more health benefits than exercising alone [2]. Further investigation is needed to clarify sex-specific determinants to increase participation in sports groups among men.

This study examined performance-based physical functions as determinants of participation in sports groups using a health checkup in a venue-based survey, and this has not been reported previously. Surprisingly, physical functions such as muscle strength and gait speed were not associated with new participation in sports groups. This finding suggested that older adults joined sports groups regardless of their level of physical function. A previous study showed that physical functions, such as gait speed, were associated with moderate- to vigorous-intensity physical activity [28]. There are various types of sports groups for older adults in the community in Japan, such as fitness exercises, weight exercises, walking, golf, dance, and yoga [3]. Because the intensity of these sports varies widely [29], it is possible that older adults choose to participate in groups with intensity suitable to them. To examine this possibility, assessing the intensity and types of sports will be necessary in future studies. In addition, it should be noted that the participants in our study had a relatively higher physical function than the reference values for community-dwelling older Japanese [20].

Older adults who engaged in regular exercise at BL were two times more likely to participate in sports groups after a year than those who did not engage in regular exercise. This result was plausible because these individuals may already have had a positive attitude toward sports and a readiness to participate in sports groups. According to the self-determination theory, intrinsic motivation is considered the highest self-determined motivation in the field of exercise promotion [30]. The high level of motivation for physical activity and exercise may have led to participation in sports groups. Furthermore, in extrinsic motivation in self-determination theory, external regulation (motivation by the recommendation of others) is the lowest degree of self-determination, while integrated regulation (motivation by conformity to one’s own values) and identified regulation (motivation by the conviction that it is necessary for oneself) are high degrees of self-determination [30]. Therefore, when promoting participation in sports groups, it is considered effective to explain the effects of participation in the group on one’s health to convince people that it is necessary for them and to work on their values, rather than simply encouraging them to participate. However, attempting to establish a habit of regular exercise to promote participation in sports groups is not reasonable because convincing people to exercise regularly per se is difficult. In Japan, more than half of older adults do not exercise regularly [19]. Globally, one in four adults do not meet the recommendations for physical activity, which is a concept that includes exercise [31]. Convincing people to be active is a global challenge. To accelerate participation in sports groups among regularly exercising older adults, it may be effective to build a platform for introducing them to groups that perform the sports that they are currently doing alone.

Having a larger social network was found to be a significant determinant of new participation in sports groups. This result was in line with that reported by Yamakita et al. in their cross-sectional study [15]. In their study, the frequency of meeting friends, number of met friends, receiving and providing emotional support, and participation in other types of group activities were strongly associated with participating in sports groups. They reported that social integration, including social support and contact with friends, could positively affect participation in sports groups because social networks affect healthy lifestyles. However, because of the study’s cross-sectional design, it was unclear whether participation in sports groups expanded social relationships or whether having fulfilling social relationships led to participation in sports groups. The present study revealed this causal relationship owing to its longitudinal design. In the area of physical activity and exercise, it is well known that interventions through social networks are effective in promoting physical activity in the community. Peck et al. reported that word of mouth from friends or relatives was the most effective method for recruiting inactive women to a community-based physical activity program compared with mass media campaigns [32]. Another community-wide intervention that employed network intervention and promoted encouragement by community leaders successfully promoted physical activity among community-dwelling middle-aged and older adults [33]. According to a review conducted by Valente et al., using opinion leaders was one of the most frequent strategies in a network intervention aimed at promoting behavior change toward healthy lifestyles [34]. The results of the present study suggested that interventions through social networks may also be effective in encouraging new participation in sports groups. However, the OR is less than 1.68 for social networks and the effect size is very small [35].

Although self-rated health and social cohesion were not associated with new participation in sports groups in this study, an earlier cross-sectional study found associations between these variables [15]. These differences may depend on differences in study design. In other words, it is possible that participation in sports groups resulted in higher self-rated health and social cohesion, rather than older adults with higher scores on these factors participating in sports groups. Cognitive function is considered a factor that influences social participation [36], but no association was found in this study. This may be due to the high MMSE scores of the participants in this study.

This study has some limitations. First, we were unable to examine important factors related to physical activity, such as individual willingness to engage in sporting activity. Second, we were unable to define what constitutes a sport group or examine the types of sports groups. Third, we may have underestimated the results because of the low follow-up rate. Finally, as the study was carried out among community-dwelling older adults in an urban area of Japan, the findings may not be generalizable to other areas with different cultures. Nevertheless, the results of this study provide an insight into the factors that influence the participation of older adults in sports groups, which may be used to inform the development of strategies to promote increased participation. Additionally, the prospective design was an important strength of this study.

## Conclusions

Encouraging older adults to participate in sports groups could become one of the most important interventions to promote health in an aging society. We found a sex difference in new participation in sports groups. We showed that the determinants of new participation in sports groups were lifestyle habits and social factors, rather than physical functions. Interventions focusing on these factors, for example via social networks, may be effective in encouraging new participation in sports groups among older adults, which, in turn, will facilitate healthy aging.
